# OsSAPK2 Confers Abscisic Acid Sensitivity and Tolerance to Drought Stress in Rice

**DOI:** 10.3389/fpls.2017.00993

**Published:** 2017-06-13

**Authors:** Dengji Lou, Houping Wang, Gang Liang, Diqiu Yu

**Affiliations:** ^1^Key Laboratory of Tropical Plant Resources and Sustainable Use, Xishuangbanna Tropical Botanical Garden, Chinese Academy of SciencesKunming, China; ^2^College of Life Sciences, University of Chinese Academy of SciencesBeijing, China

**Keywords:** SAPK2, abscisic acid (ABA), rice, CRISPR/Cas9 system, germination, drought tolerance, reactive oxygen species (ROS)

## Abstract

SNF 1-RELATED PROTEIN KINASE 2 (SnRK2) is a family of plant-specific protein kinases which is the key regulator of hyper-osmotic stress signaling and abscisic acid (ABA)-dependent development in various plants. Among the rice subclass-I and -II SnRK2s, osmotic stress/ABA–activated protein kinase 2 (SAPK2) may be the primary mediator of ABA signaling. However, SAPK2 has not been comprehensively characterized. In this study, we elucidated the functional properties of SAPK2 using loss-of-function mutants produced with the CRISPR/Cas9 system. The *SAPK2* expression level was strongly upregulated by drought, high-salinity, and polyethylene glycol (PEG) treatments. The *sapk2* mutants exhibited an ABA-insensitive phenotype during the germination and post-germination stages, suggesting that SAPK2 had a pivotal role related to ABA-mediated seed dormancy. The *sapk2* mutants were more sensitive to drought stress and reactive oxygen species (ROS) than the wild-type plants, indicating that SAPK2 was important for responses to drought conditions in rice. An additional investigation revealed that SAPK2 increased drought tolerance in the following two ways: (i) by reducing water loss *via* the accumulation of compatible solutes, promoting stomatal closure, and upregulating the expression levels of stress-response genes such as *OsRab16b*, *OsRab21*, *OsbZIP23*, *OsLEA3*, *OsOREB1* and slow anion channel (SLAC)-associated genes such as *OsSLAC1* and *OsSLAC7*; (ii) by inducing the expression of antioxidant enzyme genes to promote ROS-scavenging abilities that will ultimately decrease ROS damages. Moreover, we also observed that SAPK2 significantly increased the tolerance of rice plants to salt and PEG stresses. These findings imply that SAPK2 is a potential candidate gene for future crop improvement studies.

## Introduction

Rice (*Oryza sativa*) is a cereal that is a staple food crop for many people worldwide. The primary challenge for increasing rice production is overcoming a global water shortage, which can severely limit rice yields (Zhang, 2007). Drought stress induces various morphological and physiological changes in plants. Under drought or salinity stress conditions, H_2_O_2_ and abscisic acid (ABA) are often generated in many biological systems.

In plants, drought and salinity stresses usually cause oxidative damage via the production of reactive oxygen species (ROS), such as H_2_O_2_ and superoxide (Zhu, 2001; Mittler, 2002; Xiong and Zhu, 2002). High ROS-scavenging activities decrease the overaccumulation of ROS in plants, thereby inhibiting the onset of programmed cell death (Mittler, 2002; Farooq et al., 2009; Hou et al., 2009). Plant responses to the overaccumulation of ROS involve two mechanisms. The main ROS-scavenging mechanism depends on the activation of antioxidant enzymes, including superoxide dismutase (SOD), ascorbate peroxidase (APX), and catalase (CAT). The other mechanism relies on non-enzymatic molecular compounds such as ascorbic acid (AsA) (Jabs et al., 1996; Mittler, 2002; Xiong and Zhu, 2002; Farooq et al., 2009; Hou et al., 2009; Das and Roychoudhury, 2014).

Abscisic acid has a central role in seed dormancy, germination, and the acquisition of desiccation tolerance (Wasilewska et al., 2008). Since ABA was discovered in the 1960s, many details regarding the physiological importance of this hormone have been documented (Weiner et al., 2010). The ABA signaling pathway has been characterized as a signaling complex consisting of PYR/PYL/RCAR-type ABA receptors, the type A protein phosphatase 2Cs (PP2Cs), and sucrose non-fermenting 1-related protein kinases 2 (SnRK2s) (Fujii et al., 2009; Ma et al., 2009; Melcher et al., 2009). In the absence of ABA, the ABA signaling pathway is repressed via dephosphorylation and inactivation of SnRK2s by PP2Cs. As ABA accumulates and is perceived, RCAR/PYR receptors bind to PP2Cs and SnRK2s are released. The SnRK2s then phosphorylate downstream substrates, and activate ABA-responsive gene expression or other responses (Fujii et al., 2009; Ma et al., 2009; Park et al., 2009; Santiago et al., 2009; Umezawa et al., 2009).

Previous studies have confirmed that protein phosphorylation and dephosphorylation play crucial roles in abiotic stress responses (Sopory and Munshi, 1998; Umezawa et al., 2013). SNF 1-related protein kinases (SnRKs), which constitute a plant-specific kinase group, take their name from their fungal homolog SNF1 (AMP-activated protein kinase). In plants, the SnRK family comprises 38 members which can be subdivided into three sub-families: SnRK1, SnRK2, and SnRK3. Compelling evidence suggests that SnRK2s are involved in ABA and/or stress signaling pathways. Ten plant-specific SnRK2s have been identified in Arabidopsis thaliana (SnRK2.1-2.10) and rice (SAPK1-10) (Yoshida et al., 2002; Hrabak et al., 2003; Kobayashi et al., 2004; Halford and Hey, 2009). The ten members of SnRK2s are divided into three subclasses based on their domain structures. In *A. thaliana*, subclasses I contains *SnRK2.1*, *SnRK2.4*, *SnRK2.5*, *SnRK2.9* and *SnRK2.10*; subclasses II contains *SnRK2.7* and *SnRK2.8*; subclasses III contains *SnRK2.2*, *SnRK2.3*, and *SnRK2.6*. In *A. thaliana*, all SnRK2 except SnRK2.9 were activated by hyperosmotic stress, and five members are activated by ABA (Boudsocq et al., 2004). Three members of subclasses III, *SRK2E/OST1/SnRK2.6*, *SRK2D/SnRK2.2* and *SRK2I/SnRK2.3*, have been sufficiently studied. The *snrk2.2/2.3* double mutant, but not *snrk2.2* or *snrk2.3* single mutants, showed insensitive to ABA in seed germination and root growth inhibition (Fujii et al., 2007). The *snrk2.6* (*ost1*) mutant was insensitive to ABA and did not exhibit ABA induced stomatal closure (Mustilli et al., 2002). In the *snrk2.2/3/6* triple mutant all examined ABA responses are blocked (Fujii et al., 2009; Fujita et al., 2009). These results demonstrate that SnRK2.2, 2.3, and 2.6 are functional redundancy in ABA regulation of guard cells, seed germination and seedling growth. All members of SnRK2 subclass I, SnRK2.1/SRK2G, SnRK2.4/SRK2.4A, SnRK2.5/SRK2H and SnRK2.10/SRK2B are activated by osmotic stress, but not by ABA (Boudsocq et al., 2004). SnRK2.4 and SnRK2.10 were primarily identified in a screen for PA-binding proteins (Testerink et al., 2004). McLoughlin et al. (2012) proved that SnRK2.4 and SnRK2.10 were rapidly activated in response to salt and involved in the maintenance of root system architecture under saline conditions. Protein kinase SnRK2.10/SRK2B was involved in drought tolerance by phosphorylating a conserved motif found in the stress-related dehydrin (LEA) proteins (Vlad et al., 2008). SnRK2.4 was shown to play a role for membrane association in the response to salt stress (McLoughlin et al., 2012). Members of SnRK2s subclass II, SRK2F/SnRK2.7 and SRK2C/SnRK2.8, were strongly activated by hyperosmotic stress but weakly activated by ABA in *A. thaliana* and the activation of SRK2C/SnRK2.8 and SRK2F/SnRK2.7 by ABA was considerably lower than that of SRK2D/E/I (SnRK2.2/2.3/2.6) (Boudsocq et al., 2004, 2007). SRK2C/SnRK2.8 positively regulated drought tolerance in *A. thaliana* roots, and overexpression of *SRK2.8*-GFP increased drought tolerance due to up-regulation of stress-responsive gene in *A. thaliana* (Umezawa et al., 2004). SRK2C/SnRK2.8 was also shown to function in metabolic processes in plant growth and overexpression of *SRK2C/SnRK2.8* enhanced plant growth in *A. thaliana* (Shin et al., 2007). Under drought stress, the wild type, *snrk2.7*, *snrk2.8*, and the *snrk2.7/2.8* double mutant, did not show any clear differences in survival or visible damage, but microarray analysis suggested that subclass II SnRK2s regulate some drought-responsive genes involving ABA responsive element binding transcription factors (AREB/ABF) and their targets (Mizoguchi et al., 2010). These studies suggest subclass II SnRK2s play important roles in drought stress signaling in *A. thaliana*.

All 10 osmotic stress/ABA–activated protein kinases (*SAPKs*), which encode SnRK2 family protein kinases in the rice genome, are activated by hyperosmotic stress, while SAPKs 8–10 are also strongly activated by ABA (Kobayashi et al., 2004). In rice, subclasses I, II, and III of SnRK2s, contain *SAPK3* through *SAPK7*, *SAPK1* and *SAPK2*, and *SAPK8* through *SAPK10*, respectively (Kobayashi et al., 2004). Many studies have demonstrated that subclasses I and III of SnRK2 family members are important for stress responses and development in rice. For example, Diédhiou et al. (2008) reported that the overexpression of *SAPK4* increased salt tolerance by changing the expression level of genes related to ion homeostasis and oxidative stress responses. Chae et al. (2007) revealed that ectopic expression of *OSRK1* (*SAPK6*) in transgenic tobacco plants resulted in reduced sensitivity to ABA during seed germination and root elongation. Nam et al. (2012) proved that the overexpression of *OSRK1* (*SAPK6*) enhanced phosphorylation activities and salt-sensitive root growth in rice. Lin et al. (2015) observed that SAPK8, 9 and 10 phosphorylated the Tiller Enhancer protein and inhibited the interaction between this protein and OsPYL10. Furthermore, SAPK9 was reported to positively regulate ABA-mediated stress signaling pathways in rice (Dey et al., 2016). Kim et al. (2012) proved that the constitutive expression of *OsPYL/RCAR5* upstream of SAPK2 leaded to an ABA-hypersensitive phenotype during seed germination and early seedling growth in rice. Recently, studies indicated that OsbZIP23 and OsbZIP46 were phosphorylated by SAPKs to increase drought stress tolerance in rice (Tang et al., 2012; Zong et al., 2016). These data indicate that SAPK2 is probably crucial for ABA-mediated abiotic stress responses and developmental controls in rice, although SAPK2 has not been fully characterized. Therefore, clarifying the specific function of SAPK2 in rice is necessary for developing abiotic stress-tolerant transgenic rice.

In this study, we used the CRISPR/Cas9 system to generate *sapk2* mutants. Expression analyses revealed that *SAPK2* expression was strongly induced by drought, NaCl, and polyethylene glycol (PEG) treatments, but not by ABA. Germination assays proved that the *sapk2* mutants exhibited an ABA-insensitive phenotype during the germination and post-germination stages. Drought tolerance assays indicated that *sapk2* mutant lines were sensitive to drought conditions, with lower survival rates than the wide-type plants. The mutants also exhibited greater water loss, lower proline and soluble sugar contents, higher proportions of fully open stomata, higher ROS levels, and lower antioxidant enzyme activities. Quantitative reverse transcription polymerase chain reaction (qRT-PCR) analyses indicated that SAPK2 improved drought tolerance by enhancing the expression of stress-responsive and antioxidant enzyme genes in rice. We also observed that SAPK2 increased the tolerance of rice to salt and PEG stresses. Overall, these results confirmed that SAPK2 was an important positive regulator of ABA-dependent abiotic stress responses and developmental controls in rice.

## Materials and Methods

### Mutant Generation Methods

We employed the CRISPR/Cas9 system to generate *sapk2* mutants. The CRISPR/Cas9 plasmid was designed according to the protocol described previously (Liang et al., 2016). Concisely, the third coding exon of *SAPK2* was selected for guide RNA design. Double-strand DNA generated by annealing the oligo pairs, and then was cloned into the pYLCRISPR/Cas9Pubi-H vector. Rice (*O. sativa L. japonica*.) was used for transformation. Then transgenetic seedlings were kept in growth chamber at 28°C under long-day conditions (14 h light/10 h dark cycles). For mutation detection, genomic DNA extracted from mutant seedlings (all plant) were used for PCR. Then PCR products (sequence is in Supplementary Table S3) were identified by comparing the 19-bp gRNA target sequences (tagttatggaatatgctgc) to the rice reference genome (sequence is in Supplementary Table S3) (Liu et al., 2015). In the T_0_ generation, we collected 20 hygromycin-resistant plants. Based on mutation detection results, we identified two independent homozygous mutant lines in the T_1_ generation, which we named *sapk2-1* and *sapk2-7*.

The primers used for CRISPR/Cas9 (U3-SAPK2-F and U3-SAPK2-R) and mutation detection (SAPK2-u3-F and SAPK2-u3-R) were listed in the Supplementary Table S1.

### Plant Growth Conditions and Stress Treatments

Two *sapk2* mutant lines (*sapk2-1* and *sapk2-7*) and wide type rice (*O. sativa L. japonica*.) were selected for the experimental works. All the experiments were performed using the seeds of same harvest and storage conditions. The sterilized seeds from different genotypes were germinated on half-strength Murashige and Skoog (MS) medium simultaneously and kept in growth chamber at 28°C under long-day conditions (14 h light/10 h dark cycles). After 1 week germination, seedlings were then transplanted into the same pot with same amount of soil for drought stress or transferred into half-strength liquid medium for PEG and NaCl treatments.

To detect the transcript level of target genes under different stresses, wide type plants were grown in the same condition for 4 weeks. Then seedlings were treated with ABA or other different abiotic stresses. We performed ABA treatment by spraying leaves with 0.1 mM ABA. For drought stress, seedlings were exposed to the air without water supply. For PEG and NaCl treatments, Seedlings were transferred into half-strength liquid medium supplemented with 25% PEG6000 (m/v) or 175mM NaCl. With these treatments, leaves were sampled at 0, 3, 6, 12, and 24 h.

### RNA Extraction and qRT-PCR Analyses

For the qRT-PCR analysis, we used the same method as described (Jiang et al., 2016). Total RNA was isolated from rice seedlings using the TriZol reagent (Invitrogen). The cDNAs were obtained by using Superscript II in accordance with manufacturer’s instructions (Invitrogen). The qRT-PCR analysis was performed using SYBR Premix Ex Taq kit (Takara).

At least three independent biological experiments were conducted (three independent samples were conducted for each experiment and three technological replications in every independent experiment). One representative result was displayed here. Gene-specific primers used in qRT-PCR analysis were listed in Supplementary Table S1.

### GUS Staining of SAPK2

For GUS reporter analysis, the putative promoters of *SAPK2* were amplified from genomic DNA using primers Promoter-SAPK2-F and Promoter-SAPK2-R (Supplementary Table S1). The fused Pro*_SAPK2_*-GUS was cloned into the P1300 vector. Samples from *sapk2* mutant lines and wide type plants were harvested before and after drought stress, GUS staining was detected as described (He et al., 2014).

### Germination and Early Seedling Growth Assays

To test seed germination, seeds from *sapk2* mutant lines and wide type plants were surface-sterilized (75% ethanol for 5min, 40% NaClO for 30 min) and subsequently were washed at least five times with sterilized water. Then sterilized seeds were planted on half-strength MS agar medium containing 0, 2, or 5 μM ABA. Germination was recognized as complete when the coleoptiles were 5 mm long according the reported protocol (Lin et al., 2015). The germination rates were assessed at designated time (i.e., 0, 36, 48, 60, 72, 84, 96, 108, and 120 h). To test the growth performance at post-germination stage, the seeds of *sapk2* mutant lines and wide type plants were germinated on half-strength MS agar medium simultaneously. After 3 days, the seedlings were transferred to half-strength MS agar medium supplemented with 0, 2, or 5 μM ABA for further growth. Twenty seedlings were used to measure the shoot and root length. All these experiments were repeated three times, with 30 seeds per sample. Three independent experimental replications were conducted.

### Drought Tolerance Assays

After germinated simultaneously on half-strength MS agar medium for 7 days, 4-week-old seedlings of *sapk2* mutant lines and wide type plants were transplanted into the same pot with the same amount of soil. The drought phenotype was identified after withdrawing water for 7 days and re-watering for 7 days. Three independent experiments repeated at the same time and a representative result was displayed. Three independent experimental replications were conducted.

### Analyses for Water Loss Rates and Electrolyte Leakage, Content of Proline (Pro), Soluble Sugar and Malondialdehyde (MDA), Staining of Trypan Blue, 3, 3′-Diaminobenzidine (DAB), and Nitroblue Tetrazolium (NBT)

For the measurement of water loss rates (WLRs), detached flag leaves of *sapk2* mutants and wide type plants were placed in weighing dishes and left on the laboratory bench with light at room temperature. Fresh weight was weighed at indicated times (0–200 min). WLR was expressed as a percentage of initial fresh weight.

The relative ion leakage was checked following the method of Clarke et al. (2004) with slight modifications. Approximately 1g detached flag leaves from *sapk2* mutants and wide type plants were cut into 5 mm length and placed in test tubes containing 10 ml deionized water. The tubes were covered with plastic caps and placed in a water bath maintained at the constant temperature of 22°C for 2 h. The conductance of H_2_O was measured by conductivity meter (HORIBA TWIN COND B-173).

The content of free proline in leaves was estimated as described (Bates et al., 1973) with slight modifications. Approximately 0.5 g leaf segments from mutants and wide type plants are ground into powder with liquid nitrogen, and then homogenized with 10 ml of 3% sulphosalicylic acid in tube. Then centrifuged at 3,000 × *g* for 20 min and collect the supernatant. 2 ml of supernatant was reacted with 2 ml acid ninhydrin and 2 ml glacial acetic acid in a test tube at 100°C for 1 h. Following cooled on ice. And then the absorbance at 520 was measured by spectrophotometer. L-Pro was used as a standard to calculate the proline concentration.

Total soluble sugar content in leaves was determined using anthrone reagent (Zhang and Huang, 2013). Approximately 0.5 g dried leaf segments from mutants and wide type plants were ground into powder with liquid nitrogen, and then homogenized with 2 ml 80% ethanol in shaker at 200 rpm in 50 ml tube for 1 h. Following centrifuge at 6,000 × *g* for 10 min, and then transfer as much supernatant as possible into a new 5 ml tube. Add equal volume of chloroform, completely mix, and then centrifuge at 12,000 × *g* for 10 min. The aqueous part is transferred to a new tube, 50 μl of each is mixed with 4.95 ml anthrone reagent and then boiled for 15 min. Measure the optical density of glucose standards at 620 nm by spectrophotometer.

The content of MDA in leaves was detected as described (Heath and Packer, 1968) with slight modifications. Approximately 1g leaf segments from mutants and wide type plants were homogenized in 10 ml of 10% trichloroacetic (v/v) and centrifuged at 5,000 × *g* for 10 min. Following 2 ml of supernatant was reacted with 2 ml thiobarbituric acid in a test tube at 100°C for 15 min, quickly cooled on ice, and the absorbance at 532 was measured by spectrophotometer. The MDA content was expressed as nmol g^-1^ FW.

Trypan Blue, DAB and NBT Staining were performed as described (Jiang et al., 2016).

For the above assays, consistent results were obtained from at least three experiments, and the result from one experimental was exhibited here. Average of three replicates was calculated to represent each data point.

### Analyses of Rice Stomata

Leaf samples from *sapk2* mutant lines and wide type plants (harvested before and after drought stress) were fixed by white nail polish blotting. The stomata conductance was measured following the described method (Dey et al., 2016) with modifications. The stomatal pictures were obtained using a laser confocal microscope (Nikon A1, Japan), Percentages of three levels of stomata (completely open, partially open and completely closed stomata) in *sapk2* mutants and wild type plants were calculated before and after drought stress. (*n* = 98 stomata for *S2-1*, *n* = 95 stomata for *S2-7*, *n* = 98 stomata for wild type plants).

Three independent experiments repeated at the same time and a representative result was displayed. Three independent experimental replications were conducted.

### Oxidative Enzyme Activity Measurement

The activities SOD, POD and CAT were performed as described (Jiang et al., 2016) with slight modifications. The 4-week-old seedlings of mutants and wide type plants were withheld water for 7 days, and then leaf segments (0.5 g) from control and dehydration lines were homogenized using a chilled mortar and pestle in 5 mL of 50 mM sodium phosphate buffer (pH7.8) supplemented with 1% polyvinylpyrrolidone and 10mM β-mercaptoethanol in an ice-cold mortar. After centrifugation (13,000 × *g*) for 15min at 4°C, transfer as much supernatant as possible into a new 5 ml tube. The supernatant was used to identify SOD, POD and CAT activity levels. Enzymes activities of the SOD, peroxidases (POD) and CAT were shown by U/mg protein.

Total SOD activity in the supernatant was assayed by measuring the oxidation of epinephrine at 475 nm by observing increase in absorbance monitored using spectrophotometer.

The supernatant was used for POD assay by measuring the oxidation of guaiacol to tetra guaiacol at 470 nm by observing increase in absorbance monitored. A 0.01 absorbance increase per min at 470 nm was defined as one unit of POD activity.

The supernatant was used for CAT assay by measuring the decomposition of H_2_O_2_ at 240 nm by observing decrease in absorbance using spectrophotometer.

The content of AsA was detected using commercial assay kits purchased from Nanjing Jiancheng Bioengineering Institute (Nanjing, China). The 4-week-old seedlings of mutants and wide type plants withheld water for 7 days. According to the manual, leaf segments (0.5 g) from control and dehydration lines were homogenized using a chilled mortar and pestle in 5 mL of 5% (w/v) m-phosphoric acid. Following centrifugation at 10,000 × *g* for 15 min at 4°C, the supernatant was used for the determination of AsA. Following 2 ml of supernatant was reacted with specified dosage of R1, R2, R3, and R4 (According to the manual) in a test tube at 22°C for 30 min, quickly cooled on ice, and the absorbance at 536 was measured.

The data points which presented activities of enzymes were the average of three replicates. At least three experiments were performed, and the results were consistent. The result from one set of experiments was presented here.

### Salt and Osmotic Tolerance Assays

The sterilized seeds of *sapk2* mutants and wide type plants germinated on half-strength MS medium simultaneously for 3 days. Seedlings were then transplanted in half-strength liquid MS medium for 4 weeks. For NaCl and PEG tolerance assays, Seedlings were then transferred into half-strength MS liquid medium supplemented with 175mM NaCl or 25% PEG6000 (m/v) for 3 days. After 3 days treatment, the seedlings were transferred into fresh half-strength MS liquid medium to recover for 7 days.

For NaCl and PEG treatments, the *sapk2* mutant plants and wide type plants were transplanted in the same pot. Three independent experiments repeated at the same time and a representative result displayed here. Three independent experimental replications were conducted.

### Statistical Analyses

The experiments were arranged in a completely randomized design with at least three replicates for each treatment. Excel 2010 was used for making charts. Significant difference among different treatments was identified by an analysis of variance by using SigmaPlot10.0. The data represent mean ± standard error (SE) of three independent experiments.

## Results

### *SAPK2* Expression Profile and Protein Localization

**FIGURE 1:**
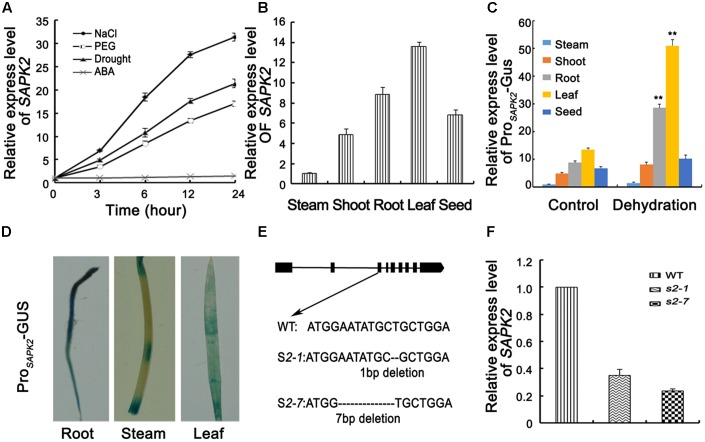
Expression analyses of *SAPK2* and Mutation Detection. **(A)** Analyses of *SAPK2* expression level under drought, NaCl, ABA, and PEG treatments. The expression level was assessed by qRT-PCR. **(B)** Expression level of *SAPK2* in different tissues by qPCR analyses. **(C)** Expression level of Pro_SAPK2_-GUS in response to drought in different tissues by qPCR analyses. **(D)** GUS staining of Pro_SAPK2_-GUS in different tissues expressing the GUS reporter gene under the control of *SAPK2* promoter. **(E)** Diagram showing that sgRNAs targets of *SAPK2* were used in the CRISPR/Cas9 system to generate *sapk2* mutants. Two independent *sapk2* mutant lines with different deletions were obtained. **(F)** Accumulation of *SAPK2* transcripts in *sapk2* mutants and wide type plants. Transcript accumulation was assessed by qRT-PCR. Error bars indicate the SD of three biological replicates. ^∗∗^indicate statistically significant differences between mutant lines and wild type plants (*P* < 0.01).

SAPK2 is a member of the rice SnRK2 II subfamily. The *SAPK2* open reading frame consists of 1,020 bp and encodes a 339-amino acid polypeptide (annotation identified in Introduction to the Rice Genome Annotation Project. database: LOC_Os07g42940^1^). We analyzed the *SAPK2* expression profiles under different stress conditions to clarify the physiological functions of SAPK2. The qRT-PCR analyses used to determine the *SAPK2* transcriptional responses to various abiotic stresses revealed that *SAPK2* expression was not induced by ABA (**Figure 1A**). In contrast, *SAPK2* expression was considerably upregulated by exposures to drought, NaCl, and PEG (**Figure 1A**).

We investigated the tissue specificity of *SAPK2* expression *via* qRT-PCR analyses using total RNA isolated from various tissues (i.e., stem, shoot, root, leave, and seed). The *SAPK2* expression level was highest in leaves, followed by the roots (**Figure 1B**). We also investigated Pro*_SAPK2_*-Gus expression in response to drought in different tissue *via* qRT-PCR analyses using total RNA isolated from various tissues (i.e., stem, shoot, root, leave, and seed). The Pro*_SAPK2_*-Gus expression level was highest in leaves, followed by roots without drought stress (**Figure 1C**). Under drought stress, the significant increase was in leaves (4.7 folds) and roots (3.2 folds) (**Figure 1C**). Tissue-specific *SAPK2* expression in response to drought was also assessed by expressing the β-glucuronidase reporter gene. Consistent with the results of the qRT-PCR analyses, β-glucuronidase signals were abundant in leaves and roots without drought stress (**Figure 1D** and **Supplementary Figure S2**). Furthermore, β-glucuronidase signals increased significantly in leaves and roots under drought stress (**Supplementary Figure S2**).

To confirm the specific SAPK2 function, we employed the CRISPR/Cas9 system to generate *sapk2* mutants, which we named *sapk2-1* and *sapk2-7*. The *sapk2-1* plants contained a 1-bp deletion in the third exon of *SAPK2*, while *sapk2-7* plants carried a 7-bp deletion in this exon (**Figure 1E**). The *SAPK2* expression level was much lower in *sapk2* mutants than in wild-type plants (**Figure 1F**). Furthermore, we also identified that the nucleotide sequences of *SAPK2* CDS from *sapk2-1* and *sapk2-7* mutant had frame shift mutation. We found that amino acid sequences of SAPK2 predicted based on these nucleotide sequences are only 106 and 104 in *sapk2-1* and *sapk2-7*, respectively (Supplementary Table S2) caused by premature termination. A previous study revealed that Ser-158, Thr-159, and Thr-162 were essential for SAPK1 activity or activation (Kobayashi et al., 2004). We considered Ser-158, Thr-159, and Thr-162 are also essential for activity or activation of SAPK2, because the kinase domains of SAPK1 and SAPK2 are highly conserved (92% amino acid identity). These results indicate that the *sapk2-1* and *sapk2-7* mutant lines lacked SAPK2.

### SAPK2 Affects Seed Germination and Early Seeding Growth in an ABA-Dependent Manner

Abscisic acid is crucial for preventing germination and inhibiting early seedling growth (Finkelstein et al., 2002). Because SAPK2 influences the ABA signal transduction pathway in rice (Kim et al., 2012), we tested the ABA sensitivity of *sapk2* mutant lines during the germination and post-germination stages.

**FIGURE 2:**
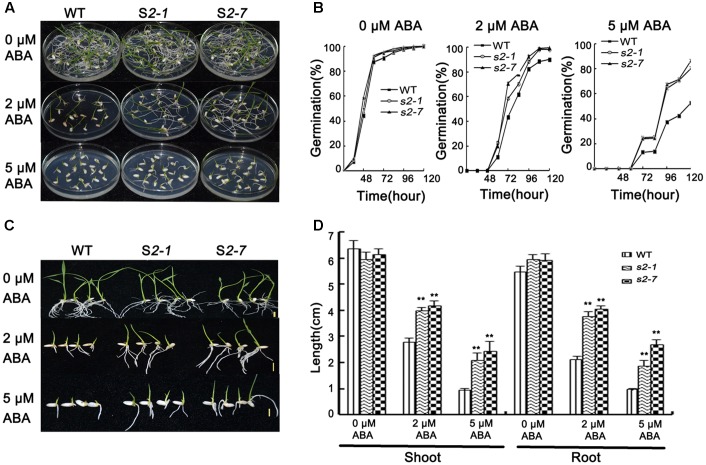
Abscisic acid sensitivity assays. **(A)** Germination phenotype of seeds from *sapk2* mutants and wide type plants grown on MS agar medium containing 0, 2, or 5 μM ABA at 5 day after imbibition. **(B)** Germination rates corresponding to **(A)**. **(C)** Growth performance of seedlings from *sapk2* mutants and wide type plants grown on MS agar medium containing 0, 2, or 5 μM ABA during the post-germination stage. **(D)** Shoot and root length corresponding to **(C)**. Error bars indicate the SD of three biological replicates. ^∗∗^indicate statistically significant differences between mutant lines and wild type plants (*P* < 0.01).

For the germination assay, seeds from two independent mutant lines (i.e., *sapk2-1* and *sapk2-7*) and wide-type plants were simultaneously sown on half-strength MS agar medium containing 0, 2, or 5 μM ABA. The germination rate was calculated at specific time points after seeds were imbibed. In the absence of ABA, there was no significant difference between *sapk2* mutants and wide-type plants (**Figures 2A,B**). However, the germination rates for the samples treated with 2 μM ABA were about 90% for the wild-type seeds and nearly 100% for the *sapk2* mutant lines (i.e., the *sapk2-1* and *sapk2-7* seed germination rates were about 98 and 99%, respectively). At 5 μM ABA, the germination rates of *sapk2* mutant lines were significantly higher than that of wide-type seeds (i.e., about 81 and 87% of *sapk2-1* and *sapk2-7* seeds germinated, while only about 52% of wild-type seeds germinated) (**Figures 2A,B**). These results indicated that the seeds of *sapk2* mutant lines were mostly insensitive to ABA during the germination stage.

The insensitivity of *sapk2* mutant lines to ABA was also confirmed during the post-germination stage. Wild-type and *sapk2* mutant seeds were simultaneously germinated on half-strength MS agar medium for 3 days, and then seedlings were transferred to the same medium supplemented with 0, 2, or 5 μM ABA. Shoot and root lengths were measured 7 days after seedlings were transferred. There was no significant difference between wild-type and *sapk2* mutant plants when the medium lacked ABA. In contrast, in media with 2 or 5 μM ABA, the *sapk2* mutants had significantly longer roots and shoots than wild-type plants (*P* < 0.01) (**Figures 2C,D**). These findings suggest that *sapk2* mutant lines are relatively insensitive to ABA after the germination stage.

These results imply that SAPK2 is a positive regulator of ABA-dependent germination and post-germination development, and is required for optimal seed germination.

### SAPK2 Is Important for Dehydration Tolerance

**FIGURE 3:**
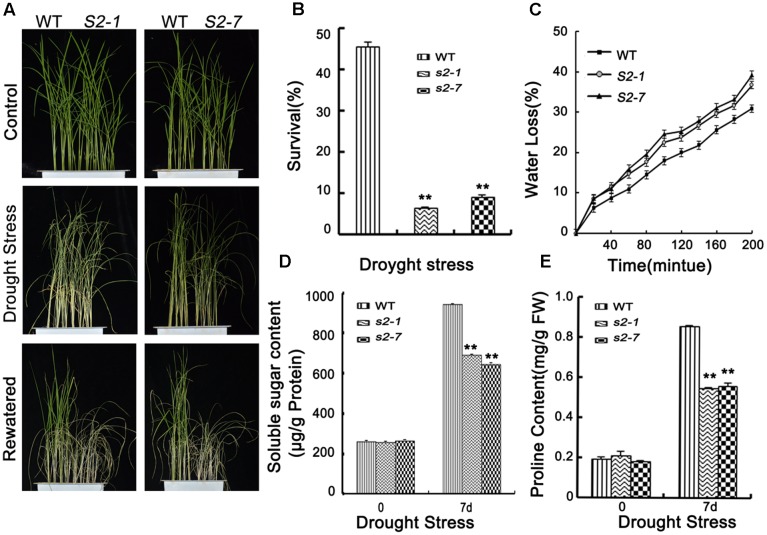
Drought stress tolerance assays. **(A)** Phenotype of *sapk2* mutants and wild-type plants before drought stress, after drought stress and re-watered for 7 days. Drought stress analysis was repeated three times. In each repeated experiment, at least 40 plants were used for each individual line. One representative picture was shown. **(B)** Survival rates corresponding to **(A)**. **(C)** Water loss rates in the leaves segregated from *sapk2* mutants and wild type plants under normal conditions. **(D)** The contents of soluble sugar and **(E)** proline in leaf tissues sampled from *sapk2* mutants and wild-type plants before and after drought stress. Error bars indicate the SD of three independent experiments. ^∗∗^indicate statistically significant differences between mutant lines and wild type plants (*P* < 0.01). FW, fresh weight.

The qRT-PCR data revealed that *SAPK2* expression was strongly induced by drought stress (**Figure 1A**). Thus, we examined the drought stress responses of SAPK2. Four-week-old plants were exposed to drought conditions by withholding water for 7 days. The survival rate (%) was determined after a 7-day rewatering period. After the 1-week drought treatment, the dehydration symptoms were slighter in wild-type plants than in *sapk2* mutants (**Figure 3A** and **Supplementary Figure S1**). When plants were rewatered, a significantly higher survival rate was observed for the wide-type plants (45.5%) than the *sapk2* mutants (i.e., the *sapk2-1* and *sapk2-7* survival rates were only 6.5 and 9.1%, respectively) (*P* < 0.01) (**Figure 3B**).

**FIGURE 4:**
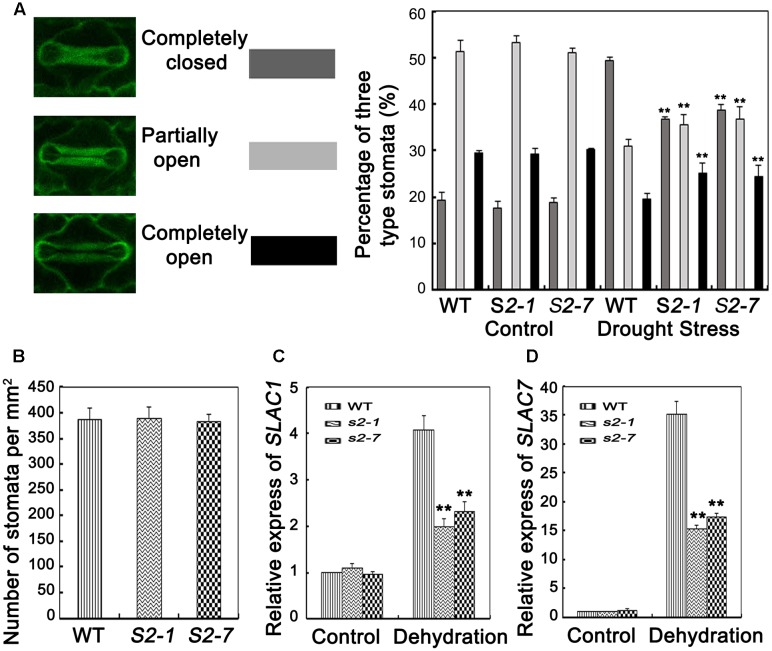
Analyses of stomata closure and relative expression level of slow anion channel (SLAC)-associated genes. **(A)** Percentages of three levels of stomata (completely open, partially open, and completely closed stomata) in *sapk2* mutants and wild type plants were calculated before and after drought stress (*n* = 98 stomata for *S2–1*, *n* = 95 stomata for *S2–7*, *n* = 98 stomata for wild type plants). **(B)** Stomata numbers per mm^2^ in 10 leaves from *sapk2* mutants and wild type plants, respectively, were counted to indicate stoma densities. **(C)** Transcript level of stomata genes *OsSLAC1* and **(D)** *OsSLAC7* in *sapk2* mutants and wild type plants before and after drought stress. Error bars indicate the SD of three independent experiments. ^∗∗^indicate statistically significant differences between mutant lines and wild type plants (*P* < 0.01).

Transpiration water loss is an important factor related to drought tolerance. Thus, we determined the leaf WLR as described by Jiang et al. (2016). We observed the leaves of both *sapk2* mutant lines lost water faster than the wild-type leaves (**Figure 3C**). Given that WLRs were higher in two mutant lines than in wide type plants, we further investigated whether the WLR was affected by the stomatal density or aperture. We then assessed the stomatal density, and determined that there were no significant difference between *sapk2* mutants and wild-type plants (**Figure 4B**). For stomatal aperture, there were also no significant difference between *sapk2* mutants and wild-type plants under normal condition (**Figure 4A**). However, under drought condition, only about 36% of stomata were completely closed in *sapk2* mutant lines (i.e., 36.7% for *sapk2-1* and 38.8% for *sapk2-7*); while nearly 50% of stomata were completely closed in wild-type plants (**Figure 4A**). Additionally, the proportions of completely open stomata for both *sapk2* mutant lines were higher than that of wild-type plants (i.e., 25.1% for *sapk2-1*, 24.5% for *sapk2-7*, and 19.6% for wild-type plants). Moreover, the percentage of partially open stomata was also higher for the *sapk2* mutant lines (i.e., 38.2% for *sapk2-1*, 36.7% for *sapk2-7*, and 30.9% for wild-type plants) (**Figure 4A**). These results suggest that SAPK2 promotes the closure of stomata result in the lower WLR, which may be critical to adapt to drought stress in rice.

To confirm our results, we also verified that *OsSLAC1* and *OsSLAC7* expression levels were significantly higher in wild-type plants than in *sapk2* mutants (**Figures 4C,D**). These results suggest that SAPK2 induces stomatal closure by upregulating the expression of slow anion channel (SLAC)-associated genes.

To clarify the mechanisms by which SAPK2 induces dehydration tolerance, we further compared the proline and soluble sugar contents of wild-type and *sapk2* mutant plants. We observed no significant difference in proline and soluble sugar contents between the wild-type and *sapk2* mutant plants under normal conditions. However, under drought stress, proline and soluble sugar contents were lower in *sapk2* mutants than in wild-type plants (**Figures 3D,E**). These results strongly suggest that SAPK2 corresponds to the accumulation of compatible solutes, such as proline and soluble sugars, in response to drought conditions in rice.

Overall, our results indicate that SAPK2 was involved in rice drought tolerance response by decreasing water loss through osmotic adjustments, stomatal closure, and upregulation of *SLAC* gene expression levels.

### Expression of Stress-Responsive Genes in *sapk2* Mutants

To comprehensively characterize SAPK2 functions in drought-stressed plants, we investigated the expression levels of several drought-related genes in *sapk2* mutants, including a pyrroline-5-carboxylate synthetase gene (*OsP5CS*), as well as *OsbZIP23*, *OsLEA3*, *OsRab16b*, *OsRab21*, *OsOREB1* and *OsDREB1A*, which all function downstream of SAPKs in the ABA-signaling pathway.

**FIGURE 5:**
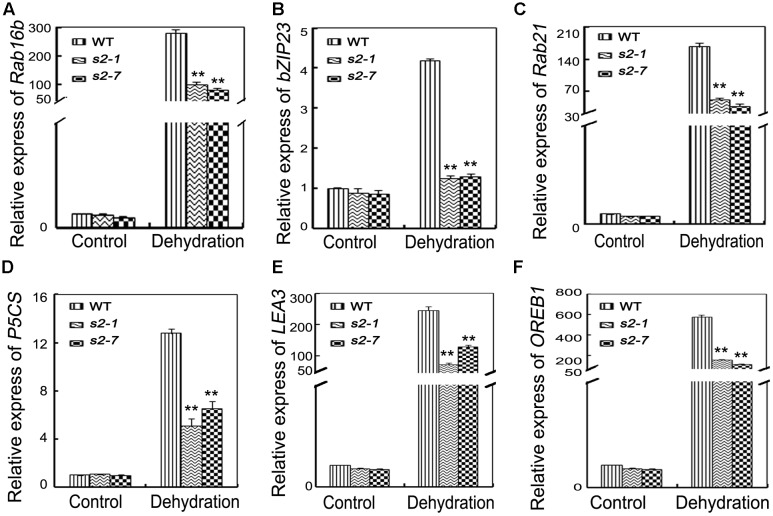
Expression of stress-response genes. **(A–F)** Relative expression levels of *OsRab16b*, *OsbZIP23*, *OsRab21*, *OsP5CS*, *OsLEA3*, and *OsOREB1* in the leaves of 4-week-old mutants and wide type plants before and after drought stress. Error bars indicate SD from three independent RNA extracts. ^∗∗^indicate statistically significant differences between mutant lines and wild type plants (*P* < 0.01).

Proline biosynthesis is catalyzed by OsP5CS. According to our qRT-PCR data, the *OsP5CS* expression level was much lower in *sapk2* mutants than in wild-type plants under drought conditions (**Figure 5D**). We also examined the expression of another five well-characterized drought resistance-related genes (i.e., *OsRab21*, *OsRab16b*, *OsbZIP23*, *OsOREB1*, and *OsLEA3*) and one control gene *OsDREB1A*. All their expression levels except *OsDREB1A* were significantly lower in *sapk2* mutants than in wild-type plants after the drought treatment (**Figures 5A–C,E,F** and **Supplementary Figure S3**). These results suggest that SAPK2 upregulate the expression of some stress-responsive genes under drought stress.

### Reactive Oxygen Species-Induced Damage in *sapk2* Mutant Plants

**FIGURE 6:**
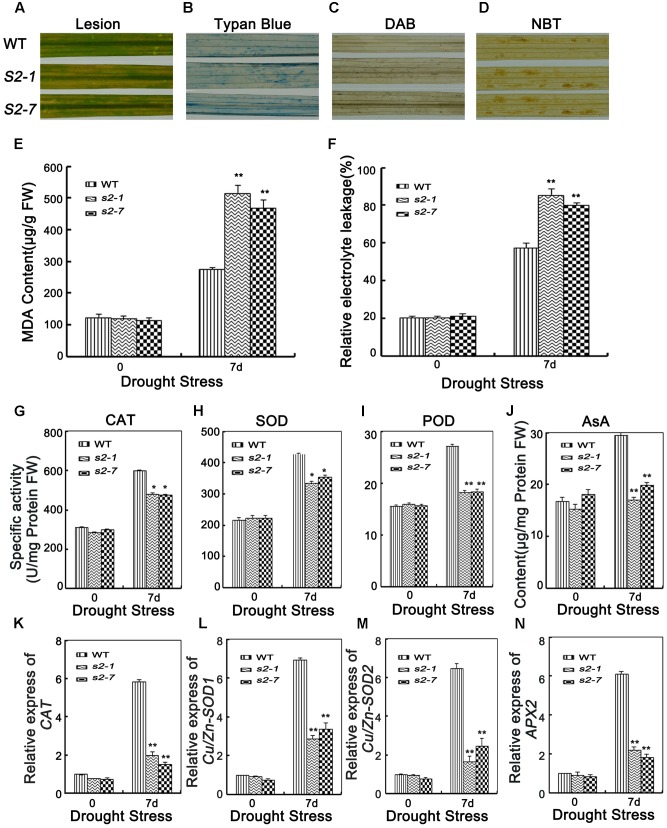
Analyses of ROS damage, ROS-scavenging ability and the expression level of oxidative enzymes genes. **(A)** Phenotype of brownish lesions after 7 days drought stress. **(B)** Phenotype of Typan blue staining after 7 days drought stress. **(C)** Phenotype of DAB staining after 7 days drought stress. **(D)** Phenotype of NBT staining after 7 days drought stress. **(E)** MDA content in the leaves of 4-week-old mutants and wide type plants before and after drought stress. FW, fresh weight. **(F)** Relative electrolyte leakage in the leaves of 4-week-old mutants and wide type plants before and after drought stress. **(G–I)** POX, SOD, and CAT activities in the leaves of 4-week-old mutants and wide type plants. **(J)** The content of AsA in the leaves of 4-week-old mutants and wide type plants. **(K)** Relative expression of oxidative enzymes genes *CAT*, **(L)** *Cu/Zn-SOD1*, **(M)** *Cu/Zn-SOD2*, and **(N)** *APX2* in the leaves of 4-week-old mutants and wide type plants. Error bars indicate SD from three independent RNA extracts. ^∗^indicate statistically significant differences between mutant lines and wild type plants (*P* < 0.05).

Because *sapk2* mutants exhibited reduced drought tolerance, we assessed whether SAPK2 affects drought tolerance *via* ROS detoxification. The leaves of wild-type and *sapk2* mutant plants began to produce brownish lesions after the 7-day drought treatment (**Figure 6A**). However, the lesions were more severe on the *sapk2* mutant leaves. This result was consistent with that of the leaf cell death analysis based on trypan blue staining (**Figure 6B**).

To detect H_2_O_2_ and superoxide damage, leaves of wild-type and *sapk2* mutant plants were stained with DAB and NBT, respectively, after 7 days of drought stress. Considerably more brown spots caused by H_2_O_2_ and superoxide were observed in the *sapk2* mutants than in the wild-type plants (**Figures 6C,D**). These results suggest that the *sapk2* mutants are unable to efficiently remove the H_2_O_2_ and superoxide produced under drought conditions.

Malondialdehyde (MDA) is the product of lipid peroxidation resulting from ROS activities and a stress-specific molecular marker indicating the extent of cellular damage (Mittler, 2002). To confirm that SAPK2 improves drought tolerance through ROS detoxification, we examined MDA contents. There were no significant differences in the MDA contents of *sapk2* mutants and wild-type plants prior to the drought treatment. Among the drought-stressed plants, the *sapk2* mutant lines accumulated more MDA than the wild-type plants (**Figure 6E**). Moreover, electrolyte leakage is widely used as an indicator of membrane damage in studies of stress-induced injuries and stress tolerance in plants (Lee and Zhu, 2010). Thus, we also measured electrolyte leakage due to drought stress, and determined that the leakage was greater in *sapk2* mutants (i.e., > 80%) than in wild-type plants (i.e., about 60%) (**Figure 6F**). These results imply that the decreased drought tolerance of the *sapk2* mutants is likely a consequence of increased cell membrane damage caused by ROS.

### Decreased ROS-Scavenging Ability and Expression of Antioxidant Enzyme Genes in *sapk2* Mutants

Given the enhanced ROS-induced damage of the *sapk2* mutants, we compared the SOD, peroxidase (POD), and CAT activities, as well as the AsA contents between *sapk2* mutants and wild-type plants. Under normal growth conditions, there were no significant differences between *sapk2* mutants and wild-type plants regarding POD, SOD, and CAT activities, and AsA contents. However, after a 7-day drought treatment, the antioxidant enzyme activities and AsA contents were significantly lower in *sapk2* mutant lines than in wild-type plants (**Figures 6G–J**). These results indicate that ROS-scavenging is inhibited in the *sapk2* mutant lines, resulting in an accumulation of ROS damage.

To confirm that in the *sapk2* mutant lines ROS-scavenging was suppressed, we assessed the expression levels of several antioxidant genes. Under drought conditions, the *OsCAT*, *OsCu/Zn-SOD1*, *OsCu/Zn-SOD2*, and *OsAPX2* transcript levels were up-regulated in wild-type and *sapk2* mutant plants, but the expression levels were significantly higher in the wild-type plants (**Figures 6K–N**). These results were consistent with the observed higher antioxidant enzyme activities in wild-type plants than in *sapk2* mutants, and further confirmed that SAPK2 limits ROS-induced damage by promoting the expression of antioxidant enzyme genes.

### Decreased Tolerance of *sapk2* Mutants to NaCl and Polyethylene Glycol

**FIGURE 7:**
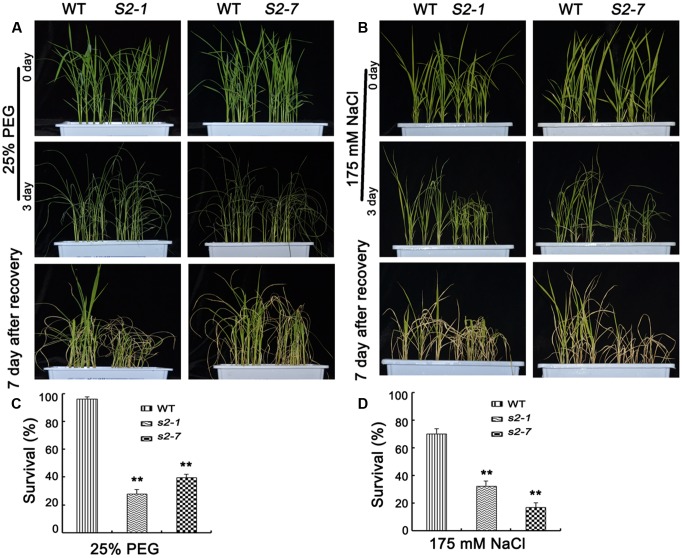
Reduced NaCl and PEG tolerance in *sapk2* mutant plants. **(A)** Phenotype before 25% PEG stress, after 25% PEG stress and recovery for 7 days of *sapk2* mutants and wild-type plants. **(B)** Survival rates corresponding to **(A)**. **(C)** Phenotype before 175 mM NaCl stress, after 175 mM NaCl stress and recovery for 7 days of *sapk2* mutants and wild-type plants. **(D)** Survival rates corresponding to **(C)**. Analysis for PEG and NaCl stress was repeated three times. In each repeated experiment, at least 40 plants were used for each individual line. One representative picture was shown here. Error bars indicate SD from three independent RNA extracts. ^∗∗^indicate statistically significant differences between mutant lines and wild type plants (*P* < 0.01).

Considering *SAPK2* expression was strongly induced by NaCl and PEG (**Figure 1A**), we explored SAPK2 functions in plants treated with NaCl or PEG. For the PEG tolerance assays, we observed the phenotypes of *sapk2* mutants and wild-type plants grown in half-strength liquid MS medium supplemented with 25% PEG-6000 for 3 days. Survival rates were calculated after plants were allowed to recover in fresh half-strength liquid MS medium for 7 days. The *sapk2* mutant plants exhibited a more severe PEG stress phenotype, with a much lower survival rates than wild-type plants (i.e., 96.3% for wild-type plants, 28.1% for *sapk2-1*, and 39.6% for *sapk2-7*) (**Figures 7A,B**). For the NaCl stress assays, we observed the phenotypes of *sapk2* mutants and wild-type plants grown in half-strength liquid MS medium containing 175 mM NaCl for 3 days. The survival rates were assessed after plants recovered in fresh half-strength liquid MS medium for 7 days. The *sapk2* mutants were more badly damaged by salt stress, and had significantly lower survival rates than the wild-type plants (i.e., survival rates: 70.4% for wild-type plants, 32.6% for *sapk2-1*, and 17% for *sapk2-7*) (**Figures 7C,D**). These results suggested that the loss of function of SAPK2 decreased osmotic stress tolerance such as NaCl, PEG and drought stress in rice.

## Discussion

Known as crucial positive regulators of ABA and osmotic stress responses in *A. thaliana* and rice, SnRK2s play important roles in various processes. SAPK2 is a member of SnRK2 subclass II in rice. However, little information is available regarding the functional properties of rice SAPK2. In this study, we characterized the functional properties of SAPK2 by developing loss-of-function mutants using the CRISPR/Cas9 system.

A previous study revealed that ABA does not upregulate the expression of *SAPK2* or activate the encoded protein (Kobayashi et al., 2004). We confirmed that *SAPK2* expression was not induced by an ABA treatment. Researchers have determined that OsPYL/RCAR5, functioning upstream of SAPK2 with mechanisms similar to *A. thaliana* PYL/RCARs, modulates germination and early seedling growth in rice (Kim et al., 2012). This suggests that SAPK2 is crucial for ABA-dependent developmental controls in rice. Therefore, we characterized the ABA sensitivity of *sapk2* mutant lines regarding developmental controls. The *sapk2* mutant plants exhibited significantly decreased sensitivity to ABA during the germination and post-germination stages (**Figure 2**). These results imply that SAPK2 has a significant role in the ABA signaling pathway during the initial development stages in rice, which is very different from the function of subclass II SnRK2 in *A. thaliana*. Because subclass II SnRK2 is not so important for ABA signaling compared to subclass III in *A. thaliana*.

*SRK2C/SnRK2.8*, which is homologous to rice *SAPK2*, was expressed abundantly in roots and weakly in leaves (Umezawa et al., 2004). This tissue-specific localization is consistent with the results that SRK2C/SnRK2.8 is a positive regulator of drought tolerance in *A. thaliana* roots. However, our data indicate that the expression level of *SAPK2* is highest in leaves, followed by the roots (**Figures 1B–D**), which is consistent with the expression patterns observed for other drought- and salt-tolerance genes as well as other *SnRK2* family members (Xiang et al., 2007, 2008; Mao et al., 2010; Tian et al., 2013; Dey et al., 2016). This tissue-specific expression pattern further suggests that SAPK2 may function similar to SAPK8 or SAPK9 in rice when plants exposed to osmotic stress, possibly by regulating the stomatal closure (Dey et al., 2016; Sun et al., 2016).

Our results revealed that *SAPK2* expression was strongly induced by drought, NaCl, and PEG (**Figure 1A**) in rice, implying that SAPK2 may have critical roles related to osmotic stress tolerance. Mizoguchi et al. (2010) observed that *snrk2.8* mutant did not show any clear differences in survival or visible damage compare with WT under drought stress. However, we observed that *sapk2* mutant plants were sensitive to drought stress, with relatively low survival rates and high WLRs (**Figures 3A–C**). The increased WLR is not favorable for drought tolerance in the *sapk2* mutant lines. In response to drought stress, stomata often close to limit water loss by transpiration. Previous studies confirmed that *A. thaliana* OST1/SnRK2.6 and rice SAPK8 and SAPK9 affect ABA-regulated stomatal movements (Mustilli et al., 2002; Dey et al., 2016; Sun et al., 2016). Additionally, OsSLAC1 and OsSLAC7 have recently been identified as stomatal proteins involved in anion transport (Fan et al., 2015; Sun et al., 2016). We observed that, under drought stress, *sapk2* mutants had larger proportions of completely and partially open stomata than wild-type plants (**Figure 4A**). Furthermore, the *OsSLAC1* and *OsSLAC7* expression levels were significantly higher in wild-type plants than in *sapk2* mutants after drought stress treatment (**Figures 4C,D**). Thus, SAPK2 may be responsible for activating FABA-induced stomatal closure, similar to how SAPK8 functions as an essential activator of OsSLAC1 *via* phosphorylations (Sun et al., 2016). This possibility should be investigated in a future study. By contrast, SRK2C/SnRK2.8 functioning very different from SAPK2. The water-loss levels of *srk2c* mutants were similar to that of WT, and overexpression of *SRK2C/SnRK2.8* did not affect stomatal regulation (Umezawa et al., 2004). Such limited phenotype of SRK2C/SnRK2.8 may be due to tissue-specific localization of SRK2C in root tips. These results suggest that SRK2C/SnRK2.8 may not be involved in stomatal response and mediated signaling should be quite different from SAPK2.

Plants evolved two central physiological mechanisms to decrease water loss under drought conditions: osmotic adjustment and stomatal closure. Zhang et al. (2011) confirmed that accumulated proline and soluble sugar could stabilize subcellular structures and facilitate cell recovery from damages due to abiotic stress. Our results of proline and soluble sugar content analyses (**Figures 3D,E**) suggest that the sensitivity to drought and the low survival rates of *sapk2* mutants may be because of several physiological traits, including excessive water loss and low proline and soluble sugar contents. Additionally, the significantly decreased *OsP5CS* transcript levels in *sapk2* mutants were consistent with the low proline content of these plants under drought conditions (**Figure 5D**). These results indicate that SAPK2 positive regulate rice drought tolerance by decreasing water loss through osmotic adjustments.

OREB1 is activated by SAPK2 in rice (Kim et al., 2012). OsbZIP23 is a member of bZIP transcription factor family, and confers ABA-dependent drought and salinity tolerance in rice. OsLEA3 is a late embryogenesis abundant protein that protects cell membrane structures and improves drought tolerance (Xiao et al., 2007). The transcription of *OsRab21* and *OsRab16b*, which encode dehydrin proteins, is responsive to abiotic stress treatments (Yamaguchi-Shinozaki et al., 1990). The expression of *OsDREB1A* was induced by cold and used as one control gene in our study (Dubouzet et al., 2003). In our study, except *OsDREB1A*, the transcript levels of *OsRab16b*, *OsRab21*, *OsbZIP23*, *OsLEA3*, and *OsOREB1* were significantly lower in *sapk2* mutants than in wild-type plants under drought conditions. These results imply that SAPK2 has an important role related to ABA-dependent dehydration tolerance, and may serve as a transcriptional activator of these genes in the ABA signaling pathway of rice. Moreover, there is accumulating evidence that most of the transcriptional activators are localized in the nucleus, which is where SAPK2 localizes (Kim et al., 2012).

Reactive oxygen species causes lipid peroxidation, which results in the production of MDA. High MDA levels are toxic to plant cells and cause programmed cell death (Hou et al., 2009). Major ROS-scavenging mechanisms of plants include SOD, APX, and CAT (Das and Roychoudhury, 2014). The balance between SOD and APX or CAT activities in cells is crucial for determining the steady-state level of superoxide radicals and H_2_O_2_ (Mittler, 2002). Among drought-treated plants, the *sapk2* mutants exhibited high levels of superoxide, H_2_O_2_, and cell death according to NBT, DAB, and trypan blue staining, respectively (**Figures 6A–D**). The mutants also exhibited higher MDA contents and relative electrolyte leakage (**Figures 6E,F**). Our results indicate that drought-stressed *sapk2* mutants develop lesions because of their inhibited ability to detoxify ROS, which should due to decreased SOD, POD, and CAT activities as well as low AsA contents (**Figures 6G–J**) through decreasing transcription of antioxidant enzyme genes (i.e., *CAT*, *SOD*, and *APX2*) (**Figures 6K–N**). These results confirm that SAPK2 protects plants from oxidative damages by enhancing ROS detoxification.

Our results indicate that SAPK2 also increases rice tolerance to NaCl and PEG stresses (**Figure 7**). Recent studies confirmed that the overexpression of *SAPK9* may significantly enhance drought tolerance, while also increasing grain yields in drought-stressed rice (Dey et al., 2016). Shin et al. (2007) reported that overexpression of *SRK2C/SnRK2.8* enhanced plant growth in *A. thaliana*. We observed that under normal conditions, the wild-type and *sapk2* mutant plants developed similarly, suggesting that *SAPK2* may be useful for improving crop yields under drought conditions. However, we did not analyze the effects of SAPK2 on productivity. Future studies should focus on the grain yields of mutant plants under drought conditions. The findings presented herein expand our knowledge regarding the role of SAPK2 as a key kinase in the ABA signaling pathway of rice. This increased characterization may be relevant for developing new rice cultivars with enhanced tolerance to drought and salt stress.

## Author Contributions

DL and DY designed the experiments. DL performed the experiments and wrote the manuscript. DL, HW, and GL analyzed the data and edited the article. All authors read and approved the final article.

## Conflict of Interest Statement

The authors declare that the research was conducted in the absence of any commercial or financial relationships that could be construed as a potential conflict of interest.

## Footnotes

1 http://rice.plantbiology.msu.edu/index.shtml

## Supplementary Material

**FIGURE S1 |** Replicates of drought stress experiments. **(A)** First replicate. Phenotype of *sapk2* mutants and wild-type plants before drought stress, after drought stress and re-watered for 7 days. **(B)** Second replicate. Drought stress analysis was repeated three times. In each repeated experiment, at least 40 plants were used for each individual line.

**FIGURE S2 |** GUS Staining of SAPK2. **(A)** GUS staining of Pro_SAPK2_-GUS in different tissues with or without drought stress in root, **(B)** steam, **(C)** leaf.

**FIGURE S3 |** Expression of stress-response genes *OsDREB1A*. Relative expression levels of *OsDREB1A* in the leaves of 4-week-old mutants and wide type plants before and after drought stress. Error bars indicate SD from three independent RNA extracts.
