# Supplementary figures and images for: OsSAPK2 Confers Abscisic Acid Sensitivity and Tolerance to Drought Stress in Rice
Source: Front Plant Sci. 2017 Jun 13;8:993. doi: 10.3389/fpls.2017.00993 (PMC5468418; doi:10.3389/fpls.2017.00993)

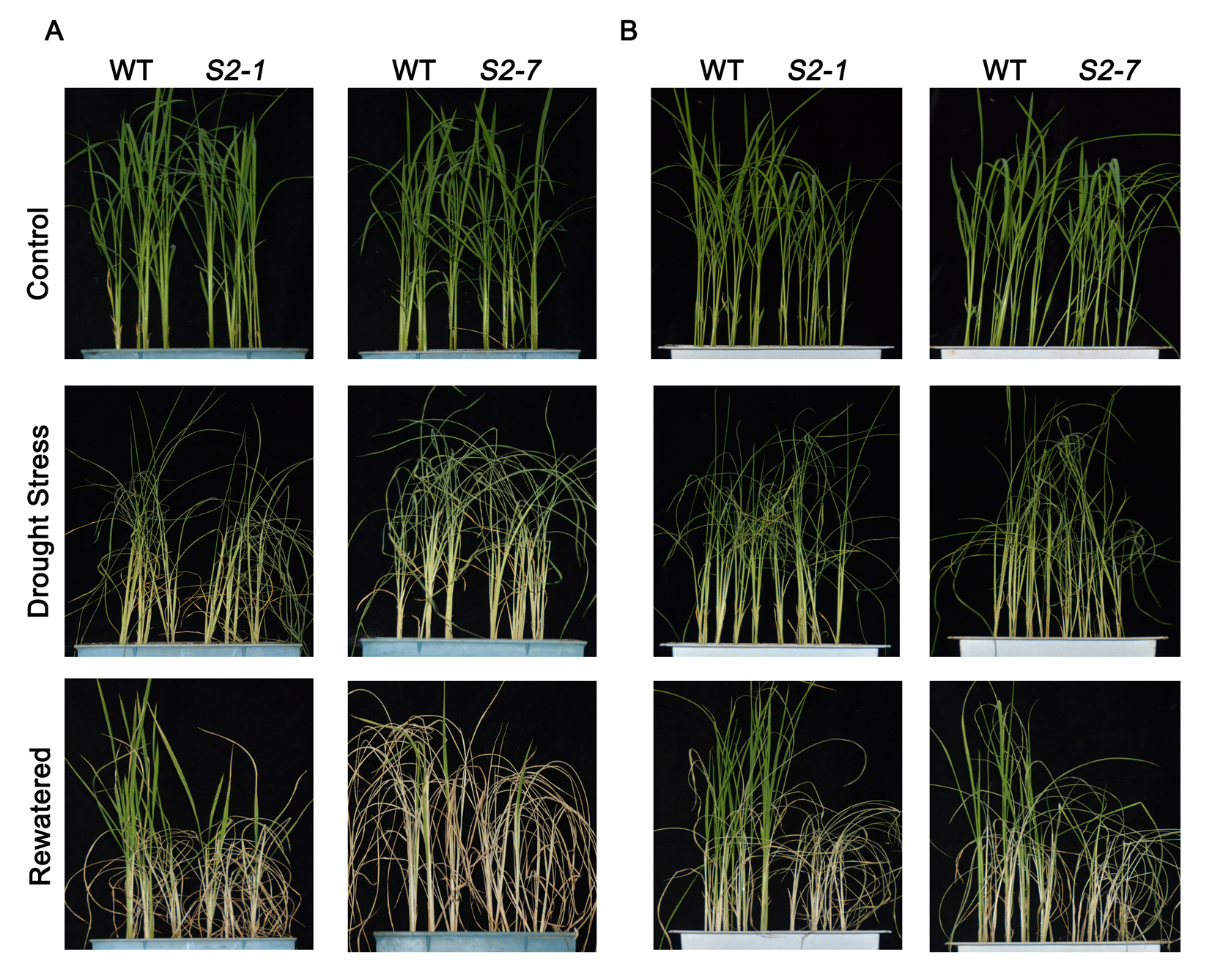

Supplement: Supplementary file 1 [file Image_1.TIF]

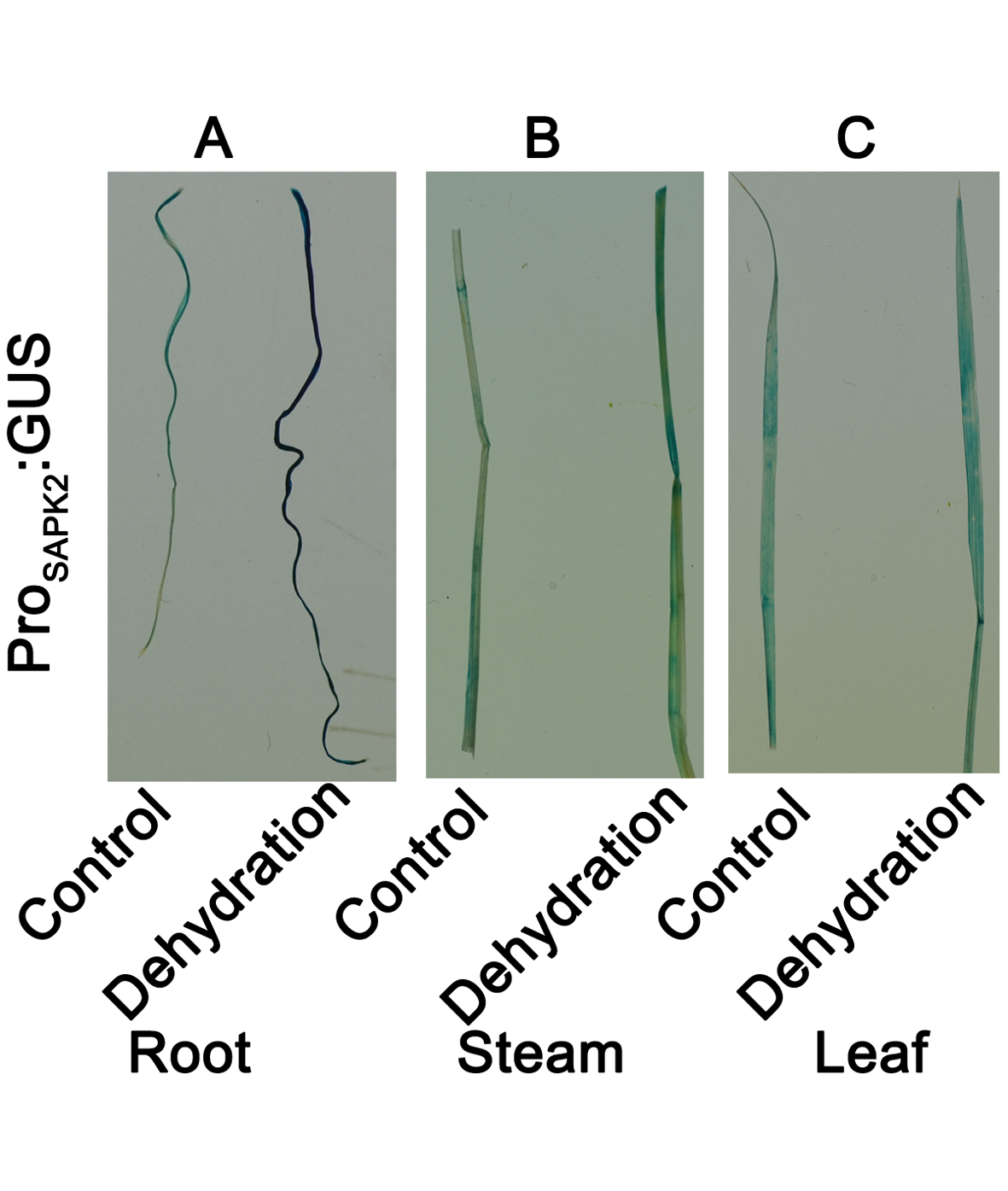

Supplement: Supplementary file 2 [file Image_2.TIF]

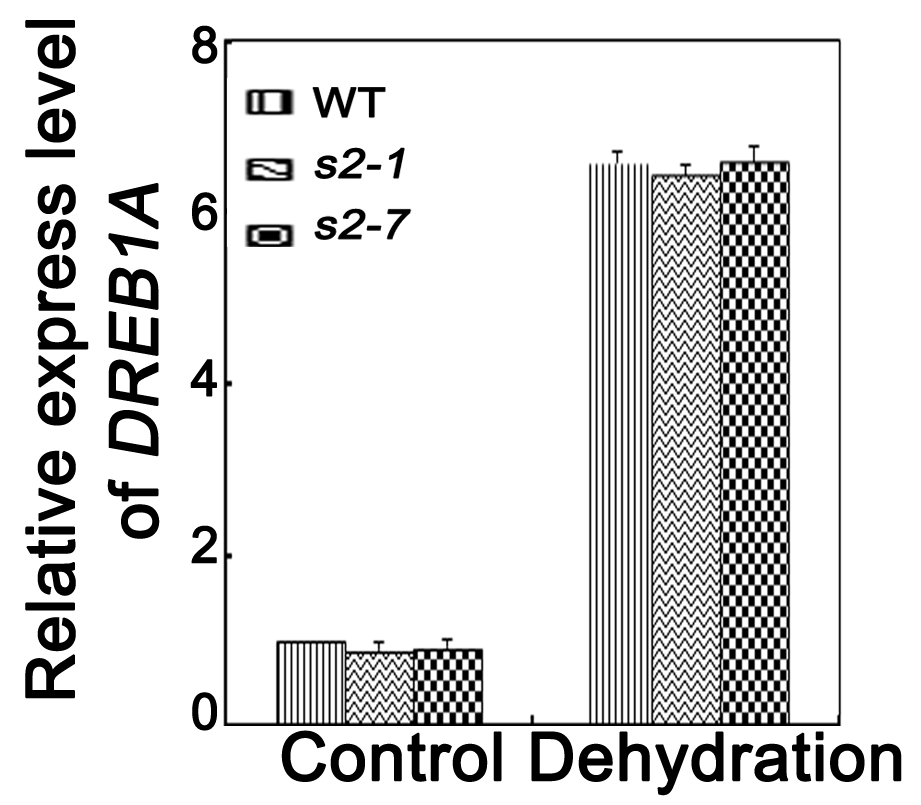

Supplement: Supplementary file 3 [file Image_3.TIF]
