# Supplementary material for: OsSAPK2 Confers Abscisic Acid Sensitivity and Tolerance to Drought Stress in Rice
Source: Front Plant Sci. 2017 Jun 13;8:993. doi: 10.3389/fpls.2017.00993 (PMC5468418; doi:10.3389/fpls.2017.00993)
Supplement: Supplementary file 4 [file Table_1.DOC]

**Supplementary Table 1. Primers and oligos used in this study**

| Name | Primer sequence (5’-3’) |
| --- | --- |
| **Plasmid Constrcutions and Mutation Detection** | |
| U3-SAPK2-F | GGCATAGTTATGGAATATGCTGC |
| U3-SAPK2-R | ATCAATACCTTATACGACGCAAA |
| SAPK2-u3-F | GGAAATGAAAGTGACCCAGCAG |
| SAPK2-u3-R | TGCTCCCATCCAAGAGAGTATTTT |
| Promoter-SAPK2-F | ATAGTCGACAAGATCCTGGGATCAAAGAAAGCTTCG |
| Promoter-SAPK2-R | ATAGGATCCCCCCACCTCCCACGACAACTCC |
| P30-SAPK2-F | ATAGGATCCATGGAGAGGTACGAGGTGATCAAGGACAT |
| P30-SAPK2-R | ATATCTAGACAATGCGCACACGAAGTCGC |
| ***qRT-PCR*** | |
| OsActin-RT-F | AGCTGCGGGTATCCATGAGA |
| OsActin-RT-R | GCAATGCCAGGGAACATAGTG |
| OsP5CS1-RT-F | GCTGACATGGATATGGCAAAAC |
| OsP5CS1-RT-R | GTAAGGTCTCCATTGCATTGCA |
| OsRab16b-RT-F | CACACCACAGCAAGAGCTAAGTG |
| OsRab16b-RT-R | TGGTGCTCCATCCTGCTTAAG |
| OsRab21-RT-F | CACACCACAGCAAGAGCTAAGTG |
| OsRab21-RT-R | TGGTGCTCCATCCTGCTTAAG |
| OsLEA3-RT-F | TGAAGAGCACGGTGGTCGG |
| OsLEA3-RT-R | GGCAGAGGTGTCCTTGTTGG |
| OsbZIP23-RT-F | GGAGCTGAACGATGAACTCCAG |
| OsbZIP23-RT-R | TCGGCTCATTCTCTCTAGAACCTC |
| OsOREB1-RT-F | AACAAGAAAGCGTCCCCACA |
| OsOREB1-RT-R | CGAGCTCCACCGTATAAGCC |
| OsDREB1A-RT-F | CTCCTACCGCACCCTCGC |
| OsDREB1A-RT-R | TAGCTCCAGAGTGGGACGTC |
| OsSLAC1-RT-F | ATCACCAAGGACAGGCAGAACG |
| OsSLAC1-RT-R | TGATGTCGTACACCCTCTTGCC |
| OsSLAC7-RT-F | TACGCTTGCAAGGTGGTCTT |
| OsSLAC7-RT-F | GGCGCGAAGAAGAAGTTGAC |
| OsCAT-RT-F | TACTTCCCATCCCGCTACGA |
| OsCAT-RT-R | TCCTTACATGCTCGGCTTCG |
| OsCu/Zn-SOD1-F | CAGGTTGAGGGAGTCGTCAC |
| OsCu/Zn-SOD1-R | GGTTGCCTCAG CTACACCTT |
| OsCu/Zn-SOD2-F | GTGAAGGCTGTTGTTGTGCT |
| OsCu/Zn-SOD2-R | GCCAGAGACACTTCCAGTCA |
| OsAPX2-RT-F | TCCTACGCCGACTTCTACCA |
| OsAPX2-RT-R | CGGCGTAATCCGCAAAGAAG |
