# Supplementary material for: OsSAPK2 Confers Abscisic Acid Sensitivity and Tolerance to Drought Stress in Rice
Source: Front Plant Sci. 2017 Jun 13;8:993. doi: 10.3389/fpls.2017.00993 (PMC5468418; doi:10.3389/fpls.2017.00993)
Supplement: Supplementary file 5 [file Table_2.DOCX]

**Supplementary Table 2** **Nucleotide sequences of *SAPK2* CDS and predicted amino acid sequences in different genotypes**

| **Genotype** | **Nucleotide sequence of *SAPK2* CDS** |
| --- | --- |
| **WT** | Atggagaggtacgaggtgatcaaggacatagggtcggggaacttcggcgtggccaagcttgtccgggatgtgcggaccaaggagctgtttgccgtcaagttcatcgagagggggcagaagatcgacgagaatgtccaaagggagattatgaaccacaggtcactgaggcatccgaacattgttagattcaaggaggttgtgctaactcccacacatttggccatagttatggaatatgctgctggaggtgagctattcgaaaggatttgcagtgctgggaggtttagcgaggatgaggcaaggttcttcttccagcagttgatttcaggagttagctactgtcattccatgcaaatatgtcatagagatttgaaactagaaaatactctcttggatgggagcatagcacctcggctcaagatatgtgattttggttactcaaagtcctctttgttgcactctcaaccgaaatctactgtcggtactccagcttatatcgctcctgaggtccttgctagaaaagaatatgatggaaaggttgctgacgtttggtcatgtggagtaactctatatgtgatgcttgttggtgcgtacccctttgaggaccctgacgaaccaagaaacttccgcaagacaattactcggatactaagcgtacaatacatggttcctgattatgttcgagtttcgatggaatgcagacatcttctgtcccggattttcgtggcaaacccagagcaacgaattaccattcctgagatcaagaaccacccatggttcctcaagaacctgccgatcgagatgactgacgagtaccagatgagcgtccagatgaacgacatcaacaccccgtcacagggcctggaggagatcatggccatcatacaggaggcgcggaagccgggtgatggctccaaattctccgggcagatcccgggcctagggagcatggagctcgacgacgttgacaccgacgacatcgacgtcgaggacagcggcgacttcgtgtgcgcattgtga |
| ***S2-1*** | Atggagaggtacgaggtgatcaaggacatagggtcggggaacttcggcgtggccaagcttgtccgggatgtgcggaccaaggagctgtttgccgtcaagttcatcgagagggggcagaagatcgacgagaatgtccaaagggagattatgaaccacaggtcactgaggcatccgaacattgttagattcaaggaggttgtgctaactcccacacatttggccatagttatggaatatgcgctggaggtgagctattcgaaaggatttgcagtgctgggaggtttagcgaggatgaggcaaggttcttcttccagcagttgatttcaggagttagctactgtcattccatgcaaatatgtcatagagatttgaaactagaaaatactctcttggatgggagcatagcacctcggctcaagatatgtgattttggttactcaaagtcctctttgttgcactctcaaccgaaatctactgtcggtactccagcttatatcgctcctgaggtccttgctagaaaagaatatgatggaaaggttgctgacgtttggtcatgtggagtaactctatatgtgatgcttgttggtgcgtacccctttgaggaccctgacgaaccaagaaacttccgcaagacaattactcggatactaagcgtacaatacatggttcctgattatgttcgagtttcgatggaatgcagacatcttctgtcccggattttcgtggcaaacccagagcaacgaattaccattcctgagatcaagaaccacccatggttcctcaagaacctgccgatcgagatgactgacgagtaccagatgagcgtccagatgaacgacatcaacaccccgtcacagggcctggaggagatcatggccatcatacaggaggcgcggaagccgggtgatggctccaaattctccgggcagatcccgggcctagggagcatggagctcgacgacgttgacaccgacgacatcgacgtcgaggacagcggcgacttcgtgtgcgcattgtga |
| ***S2-7*** | Atggagaggtacgaggtgatcaaggacatagggtcggggaacttcggcgtggccaagcttgtccgggatgtgcggaccaaggagctgtttgccgtcaagttcatcgagagggggcagaagatcgacgagaatgtccaaagggagattatgaaccacaggtcactgaggcatccgaacattgttagattcaaggaggttgtgctaactcccacacatttggccatagttatggtgctggaggtgagctattcgaaaggatttgcagtgctgggaggtttagcgaggatgaggcaaggttcttcttccagcagttgatttcaggagttagctactgtcattccatgcaaatatgtcatagagatttgaaactagaaaatactctcttggatgggagcatagcacctcggctcaagatatgtgattttggttactcaaagtcctctttgttgcactctcaaccgaaatctactgtcggtactccagcttatatcgctcctgaggtccttgctagaaaagaatatgatggaaaggttgctgacgtttggtcatgtggagtaactctatatgtgatgcttgttggtgcgtacccctttgaggaccctgacgaaccaagaaacttccgcaagacaattactcggatactaagcgtacaatacatggttcctgattatgttcgagtttcgatggaatgcagacatcttctgtcccggattttcgtggcaaacccagagcaacgaattaccattcctgagatcaagaaccacccatggttcctcaagaacctgccgatcgagatgactgacgagtaccagatgagcgtccagatgaacgacatcaacaccccgtcacagggcctggaggagatcatggccatcatacaggaggcgcggaagccgggtgatggctccaaattctccgggcagatcccgggcctagggagcatggagctcgacgacgttgacaccgacgacatcgacgtcgaggacagcggcgacttcgtgtgcgcattgtga |
| **Genotype** | **Predicted amino acid sequence of *SAPK2* CDS** |
| **WT** | M E R Y E V I K D I G S G N F G V A K L V R D V R T K E L F A V K F I E R G Q K I D E N V Q R E I M N H R S L R H P N I V R F K E V V L T P T H L A I V M E Y A A G G E L F E R I C S A G R F S E D E A R F F F Q Q L I S G V S Y C H S M Q I C H R D L K L E N T L L D G S I A P R L K I C D F G Y S K S S L L H S Q P K S T V G T P A Y I A P E V L A R K E Y D G K V A D V W S C G V T L Y V M L V G A Y P F E D P D E P R N F R K T I T R I L S V Q Y M V P D Y V R V S M E C R H L L S R I F V A N P E Q R I T I P E I K N H P W F L K N L P I E M T D E Y Q M S V Q M N D I N T P S Q G L E E I M A I I Q E A R K P G D G S K F S G Q I P G L G S M E L D D V D T D D I D V E D S G D F V C A L * |
| ***S2-1*** | M E R Y E V I K D I G S G N F G V A K L V R D V R T K E L F A V K F I E R G Q K I D E N V Q R E I M N H R S L R H P N I V R F K E V V L T P T H L A I V M E Y A L E V S Y S K G F A V L G G L A R M R Q G S S S S S * |
| ***S2-7*** | M E R Y E V I K D I G S G N F G V A K L V R D V R T K E L F A V K F I E R G Q K I D E N V Q R E I M N H R S L R H P N I V R F K E V V L T P T H L A I V M V L E V S Y S K G F A V L G G L A R M R Q G S S S S S * |
