# Supplementary material for: OsSAPK2 Confers Abscisic Acid Sensitivity and Tolerance to Drought Stress in Rice
Source: Front Plant Sci. 2017 Jun 13;8:993. doi: 10.3389/fpls.2017.00993 (PMC5468418; doi:10.3389/fpls.2017.00993)
Supplement: Supplementary file 6 [file Table_3.DOCX]

**Supplementary Table 3 Nucleotide sequences of *SAP*K2 in genomic DNA For mutation detection in different genotypes**

| **Genotype** | **Nucleotide sequence of *SAP*K2 in genomic DNA** |
| --- | --- |
| **WT** | ggaaatgaaagtgacccagcaggatattatagcctcttgttcaaatcttgaccgtgttaagccttgtttgcatttaacagaagccaaattatctgaaagttgcattattcaactatttgatggtagtttacattcattgttgcttttcatattgtcaaacatatatttagtagagactagctttaaggagttaggtggataattgcaggtatcttgttaatacctctgagcattagaaatggctgtcatattttctaagatttaaagctgttgtcataagattatatactcaaagagaacacttagtttttcaggttgtgctaactcccacacatttggccatagttatggaatatgctgctggaggtgagctattcgaaaggatttgcagtgctgggaggtttagcgaggatgaggtagagaatatttacaagtcatgatacttaaagattataagatgattcaattttgttgatggtttattcaacctatcgacaggcaaggttcttcttccagcagttgatttcaggagttagctactgtcattccatggtacacaataatctgaaatcaaattgttgttagcaactcaaatgctctatatcttacataatgatatattgccttattgtgtcagcaaatatgtcatagagatttgaaactagaaaatactctcttggatgggagca |
| ***S2-1*** | CTCATCTTGACCGTGTTAGCCTTGTTTGCATTTAACAGAAGCCAAATTATCTGAAAGTTGCATTATTCAACTATTTGATGGTAGTTTACATTCATTGTTGCTTTTCATATTGTCAAACATATATTTAGTAGAGACTAGCTTTAAGGAGTTAGGTGGATAATTGCAGGTATCTTGTTAATACCTCTGAGCATTAGAAATGGCTGTCATATTTTCTAAGATTTAAAGCTGTTGTCATAAGATTATATACTCAAAGAGAACACTTAGTTTTTCAGGTTGTGCTAACTCCCACACATTTGGCCATAGTTATGGAATATGCGCTGGAGGTGAGCTATTCGAAAGGATTTGCAGTGCTGGGAGGTTTAGCGAGGATGAGGTAGAGAATATTTACAAGTCATGATACTTAAAGATTATAAGATGATTCAATTTTGTTGATGGTTTATTCAACCTATCGACAGGCAAGGTTCTTCTTCCAGCAGTTGATTTCAGGAGTTAGCTACTGTCATTCCATGGTACACAATAATCTGAAATCAAATTGTTGTTAGCAACTCAAATGCTCTATATCTTACATAAGGAAATATTGCCTTATTGGGTCACCAATATGTCTTAAAAATTTGAAACTAAAAAAAATCCTCCTGATGTGG |
| ***S2-7*** | TGTCATCTTGACGTGTTAGCCTTGTTTGCATTTAACAGAAGCCAAATTATCTGAAAGTTGCATTATTCAACTATTTGATGGTAGTTTACATTCATTGTTGCTTTTCATATTGTCAAACATATATTTAGTAGAGACTAGCTTTAAGGAGTTAGGTGGATAATTGCAGGTATCTTGTTAATACCTCTGAGCATTAGAAATGGCTGTCATATTTTCTAAGATTTAAAGCTGTTGTCATAAGATTATATACTCAAAGAGAACACTTAGTTTTTCAGGTTGTGCTAACTCCCACACATTTGGCCATAGTTATGGTGCTGGAGGTGAGCTATTCGAAAGGATTTGCAGTGCTGGGAGGTTTAGCGAGGATGAGGTAGAGAATATTTACAAGTCATGATACTTAAAGATTATAAGATGATTCAATTTTGTTGATGGTTTATTCAACCTATCGACAGGCAAGGTTCTTCTTCCAGCAGTTGATTTCAGGAGTTAGCTACTGTCATTCCATGGTACACAATAATCTGAAATCAAATTGTTGTTAGCAACTCAAATGCTCTATATCTTACATAATGATATATTGCCTTATTGTGTCAGCAAATATGTCATAGAGATTTGAAACTAGAAAATACCTTGGGGGGGGGGGGGAAAAAAAAAAAAAAAAGGAA |
